# Einflussfaktoren auf die Mortalität bei Patienten mit hüftgelenknahen Frakturen an einem regionalen Traumazentrum

**DOI:** 10.1007/s00391-021-01869-9

**Published:** 2021-03-23

**Authors:** Ali Darwich, Elio Assaf, Roman Klein, Sascha Gravius, Christoph G. Wölfl, Ahmed Jawhar

**Affiliations:** 1 Orthopädisch-Unfallchirurgisches Zentrum, Medizinische Fakultät Mannheim der Universität Heidelberg, Universitätsklinikum Mannheim, Theodor-Kutzer-Ufer 1–3, 68167 Mannheim, Deutschland; 2 Klinik für Orthopädie, Unfallchirurgie und Sporttraumatologie, Marienhausklinikum Hetzelstift, Stiftstraße 10, 67434 Neustadt/Weinstraße, Deutschland; 3 Zentrum für Unfallchirurgie, Orthopädie und Handchirurgie, Klinikum Worms, Gabriel-von-Seidl-Straße 81, 67550 Worms, Deutschland

**Keywords:** Proximale Femurfraktur, Koxal, Letalität, Morbidität, Geriatire, Proximal femur fracture, Coxal, Lethality, Morbidity, Geriatric

## Abstract

**Hintergrund:**

Krankenhäuser der Grund- und Regelversorgung bzw. regionale Traumazentren spielen eine essenzielle Rolle in der Versorgung hüftgelenknaher Frakturen.

**Ziel:**

Die vorliegende Arbeit untersucht den Zusammenhang zwischen patientenbezogenen Parametern und der Klinikmortalität sowie dem Auftreten von Komplikationen bei solchen Frakturen an einem regionalen Traumazentrum.

**Methoden:**

Im Rahmen einer retrospektiven Studie wurden die Daten von allen Patienten, die über 2 Jahre an einem regionalen Traumazentrum mit einer hüftgelenknahen Fraktur aufgenommen wurden, erfasst. Eingeschlossen wurden nur Patienten älter als 60 Jahre. Die patientenbezogenen Parameter umfassten Alter, Geschlecht, Frakturlokalisation, Methode der operativen Versorgung, Operationszeitpunkt, Dauer der Operation und des stationären Aufenthalts, Bluttransfusion, Komplikationen, Komorbiditäten, Einnahme von Antikoagulanzien und Notwendigkeit einer postoperativen intensivmedizinischen Behandlung. Der Zusammenhang zwischen diesen Parametern und der Klinikmortalität sowie dem Auftreten von Komplikationen wurde untersucht.

**Ergebnisse:**

Daten von 360 Patienten mit 335 Operationen (w:m 225:110) mit einem Alter von 83 ±8 Jahren wurden erfasst. Die Klinikmortalität lag bei 7,76 % (*n* = 26) und erhöhte sich bei Alter > 85 Jahren (Odds Ratio [OR] 5,126; 95%-Konfidenzintervall [95 %-KI] 0,665–39,498; *p* = 0,1167), männlichem Geschlecht (OR 1,85 95%-KI [0,82–4,14]; *p* = 0,0555), Zeitpunkt der Operation > 24 h (OR 1,896 95%-KI [0,661–5,441]; *p* = 0,2341), ≥ 3 Vorerkrankungen (OR 10,61 95%-KI [3,681–27,501]; *p* < 0,0001), Einnahme von Antikoagulanzien (OR 6,19 95%-KI [2,69–14,24]; *p* < 0,0001) und notwendiger postoperativer intensivmedizinischer Behandlung (OR 5,9 95%-KI [2,56–13,76]; *p* < 0,0001).

**Schlussfolgerung:**

In der vorliegenden Studie fanden sich statistisch signifikante Einflüsse der Anzahl der Komorbiditäten, der Einnahme von Antikoagulanzien sowie der intensivmedizinischen Behandlung auf die Klinikmortalität bei Patienten mit hüftgelenknahen Frakturen an einem regionalen Traumazentrum.

**Zusatzmaterial online:**

Zusätzliche Informationen sind in der Online-Version dieses Artikels (10.1007/s00391-021-01869-9) enthalten.

## Abstract (en)

**Background:**

Primary care hospitals and regional trauma centers play an essential role in the treatment of hip fractures.

**Objective:**

This study investigated the relationship between patient-related parameters and in-hospital mortality as well as complications of hip fractures at a regional trauma center.

**Methods:**

In a retrospective study, data were collected from all patients > 60 years admitted over 2 years to a regional trauma center with a hip fracture. Patient-related parameters included age, sex, fracture location, method of surgical treatment, time of surgery, duration of surgery, length of inpatient stay, blood transfusion, complications, comorbidities, use of anticoagulant medication and need for postoperative intensive care treatment. The relationship between these parameters and hospital mortality as well as complications was investigated.

**Results:**

Data were collected from 360 patients undergoing 335 surgeries (f:m 225:110) with a mean age of 83 ± 8 years. The total in-hospital mortality rate was 7.76% (*n* = 26). Factors increasing in-hospital mortality included: age > 85 years (odds ratio [OR] 5.126; 95% confidence interval [CI] 0.665–39.498; *p* = 0.1167); male sex (OR 1.85 95%-CI [0.82–4.14]; *p* = 0.0555); time of surgery > 24 h (OR 1.896 95%-CI [0.661–5.441]; *p* = 0.2341); ≥ 3 comorbidities (OR 10.61 95%-CI [3.681–27.501]; *p* < 0.0001); intake of anticoagulants (OR 6.19 95%-CI [2.69–14.24]; *p* < 0.0001) and postoperative intensive care (OR 5.9 95%-CI [2.56–13.76]; *p* < 0.0001).

**Conclusion:**

In the present study a statistically significant influence of the number of comorbidities or Charlson comorbidity index, the intake of anticoagulant drugs and need for postoperative intensive care treatment on the in-hospital mortality of patients with proximal femoral fractures in a regional trauma center was found.

## Einleitung

Die proximalen hüftgelenknahen Femurfrakturen (HF) sind eine der häufigsten Erkrankungen der alten Menschen mit einer jährlichen Inzidenz von 110–130 Fällen/100.000 Einwohner und für Patienten > 65 Jahre 650–900 Fällen/100.000 Einwohner [23].

Da die operative Behandlung die Methode der Wahl ist, werden nahezu alle HF unter stationären Bedingungen operativ behandelt. Trotz der schnelleren Wiederherstellung der Mobilität stellen HF mit ihrer steigenden Häufigkeit mit zunehmender Lebenserwartung sowie den daraus resultierenden Kosten für die operative Sanierung, den Aufenthalt im Krankenhaus und die Nachbehandlung sowie die Behandlung möglicher Komplikationen eine große finanzielle Bürde für das Gesundheitssystem dar [19]. Die Überlebenden solcher Frakturen benötigen in 30 % der Fälle eine Pflegegradeinstufung oder -höherstufung, und 6 Monate nach dem stationären Aufenthalt steigt der Bedarf einer Unterstützung durch Pflegedienste oder Familie um 14 % an [5].

Abgesehen von der sozioökonomischen Seite weisen HF erhöhte Mortalitätsraten auf: Die Klinikmortalität liegt zwischen 5,5 und 8,3 %, die 1‑Monat-Mortalität zwischen 5 und 10 %, die 3‑Monate-Mortalität zwischen 10,1 und 14,5 % und die 1‑Jahres-Mortalität zwischen 15 und 23,9 % [16, 17, 20].

Daten über Mortalitätsraten an regionalen Traumazentren oder Krankenhäusern der Regelversorgung sind kaum zu finden. Die Daten der Untersuchungen in der Literatur über die Mortalität und deren beeinflussende Faktoren stammen nahezu ausschließlich aus Kliniken der Maximalversorgung bzw. Universitätskliniken oder überregionalen Traumazentren sowie aus Multizenter- und Querschnittsstudien. Krankenhäuser der Grund- und Regelversorgung bzw. regionale Traumazentren spielen aber eine essenzielle Rolle in der Krankenversorgung.

Gemäß dem Krankenhausplan des Landes befinden sich in Baden-Württemberg 13 überregionale Traumazentren und 47 regionale/lokale Traumazentren [4].

Ziel der vorliegenden Arbeit ist es, den Zusammenhang zwischen den patientenbezogenen Parametern und der Klinikmortalität der HF in einer Klinik der Grund- und Regelversorgung bzw. einem regionalen Traumazentrum zu untersuchen.

## Material und Methoden

### Patientenauswahl

Es wurden die Daten von allen Patienten > 60 Jahre, die über einen Zeitraum von 2 Jahren in einer Klinik für Orthopädie und Unfallchirurgie an einem regionalen Traumazentrum mit einer hüftgelenknahen Femurfrakturen (HF) aufgenommen worden, retrospektiv erfasst (*n* = 360) (Tab. 1). Bei den 360 erfassten Patienten wurden 335 Patienten operiert. 21 Patienten wurden konservativ behandelt, und 4 Patienten wurden aus Kapazitätsgründen weiterverlegt. Die erfassten patientenbezogenen Variablen sind in Tab. 1 zu finden.

**Table 1:** 

*Alter (Jahre)*	Mean ± SD (Range)	83 ± 8 (60–102)
	Männer	82 ± 9
	Frauen	84 ± 7
*Geschlecht*	Männlich	110/335 (33 %)
	Weiblich	225/335 (67 %)
*Seite*	Rechts	176/335 (52,5 %)
	Links	159/335 (47,5 %)
*Frakturtyp (ICD)*	Acetabulumfrakturen (S32.4)	1/335 (0,5 %)
	Schenkelhalsfrakturen (S72.00, S72.01, S72.03, S72.04, S72.05, S72.08)	162/335 (48 %)
	Periprothetische proximale Femurfrakturen (S72.10, S72.11, S72.2)	8/335 (2 %)
	Pertrochantäre Frakturen (S72.10, S72.11)	126/335 (38 %)
	Subtrochantäre Frakturen (S72.2)	32/335 (9,5 %)
	Abriss des Trochanter major (S72.10)	3/335 (1 %)
	Implantatversagen (S72.00, S72.01, S72.03, S72.04, S72.05, S72.08, S72.10, S72.11, S72.2)	3/335 (1 %)
*Zeitspanne zwischen Aufnahme und Operation*	< 24 h	262/335 (78 %)
	24–48 h	43/335 (13 %)
	> 48 h	30/335 (9 %)
*Anzahl der Komorbiditäten*	Mean ± SD (Range)	2 ± 1 (0–7)
Anzahl der Komorbiditäten pro Patient (*n*) %	0	53/335 (16 %)
	1	80/335 (23 %)
	2	90/335 (26 %)
	3	63/335 (19 %)
	4	32/335 (10 %)
	5	13/335 (4 %)
	6	3/335 (1 %)
	7	1/335 (1 %)
Art der Komorbiditäten und Anzahl der Patienten pro Komorbidität (*n*) %	Arterielle Hypertonie	209 (62 %)
	Diabetes mellitus	73 (22 %)
	Demenz	64 (19 %)
	Herzinsuffizienz, KHK	61 (18 %)
	VHF	58 (17 %)
	Hyperlipoproteinämie	42 (13 %)
	Niereninsuffizienz	32 (10 %)
	Zerebrale Ischämie mit ggf. sensomotorischen Residuen	31 (9 %)
	Bekanntes Tumorleiden	28 (8 %)
	pAVK	14 (4 %)
	M. Parkinson	12 (3,5 %)
	COPD	10 (3 %)
	Adipositas	9 (2,5 %)
	Hypothyreose	6 (2 %)
	Asthma bronchiale	6 (2 %)
	Hyperurikämie	2 (1 %)
*Charlson Comorbidity Index (CCI)*	Mean ± SD (Range)	4,8 ± 1,4 (2–10)
Charlson Comorbidity Index pro Patient	2	10/335 (3 %)
	3	37/335 (11 %)
	4	114/335 (34 %)
	5	80/335 (24 %)
	6	60/335 (18 %)
	7	21/335 (6 %)
	8	10/335 (3 %)
	9	2/335 (0,6 %)
	10	1/335 (0,4 %)
*Gerinnungshemmende Medikation*	Insgesamt	56/335 (17 %)
	Vitamin-K-Antagonisten	32/56 (57 %)
	Neue orale Antikoagulanzien	18/56 (32 %)
	Thrombozytenaggregationshemmern außer ASS	6/56 (11 %)

*SD* „standard deviation“, *pAVK* periphere arterielle Verschlusskrankheit, *COPD* „chronic obstructive pulmonary disease“, *KHK* koronare Herzkrankheit, *VHF* Vorhofflimmern, *ASS* Acetylsalicylsäure

Patienten mit Notwendigkeit der prä- resp. perioperativen Gerinnungsoptimierung (PPSB, FFP, Vitamin K o. Ä.) bei präoperativer dauerhafter Antikoagulanzientherapie wurden in das Studienkollektiv „unter Antikoagulation“ eingeschlossen, da die Gerinnungsoptimierung häufig zu einer relevanten Verzögerung im zeitlichen Verlauf bis zur operativen Versorgung führen und einen wesentlichen Einfluss auf die Mortalität bedingen. Patienten, die ausschließlich Acetylsalicylsäure (ASS) zu Primär- und Sekundärprophylaxe einnahmen, wurden aufgrund der Tatsache, dass hierdurch keine präoperative Gerinnungsoptimierung und keine Verzögerung bis zur operativen Versorgung resultiert, in das Patientenkollektiv „ohne Antikoagulation“ inkludiert. Aus der Studienannahme heraus sind keine Korrelationen zwischen der ASS-Einnahme und den erfassten Studienparametern ableitbar.

### Statistische Datenauswertung

Alle operierten Patienten wurden in die Auswertung eingeschlossen (*n* = 335). Zur Beschreibung der Patientenkollektive wurden prozentuale Anteile sowie Mittelwerte und Standardabweichungen (SD) verwendet. Die Beurteilung der Zusammenhänge wurde mit dem Chi-Quadrat- und dem Fisher’s Exact Test durchgeführt. Als Signifikanzniveau wurden 5 % gewählt. T‑Test und Mann-Whitney-U-Test wurden für normalverteilte und nichtnormalverteilte Stichproben verwendet. Anschließend wurden bei statistisch signifikanten Zusammenhängen logistische Regressionsanalysen durchgeführt. Die Ergebnisse werden in Form von Odds Ratio (OR) und 95 %-Konfidenzintervall (95 %-KI) dargestellt. Die statistischen Auswertungen wurden mit dem Programm SAS-Software® der Fa. SAS Institute (Cary, NC, USA) durchgeführt.

### Einhaltung ethischer Richtlinien

Diese Studie wurde gemäß den Grundsätzen der Deklaration von Helsinki durchgeführt und hat das positive Votum der zuständigen Ethikkommissionen erhalten (Ethikkommission II der Universität Heidelberg, Medizinische Fakultät Mannheim, Votum Nummer 2018-818R-MA, sowie Ethikkommission der Landesärztekammer Rheinland-Pfalz, Votum Nummer 2020-15195).

Die Daten dieser Studie stammen ausschließlich aus der Klinik für Orthopädie, Unfallchirurgie und Sporttraumatologie am Marienhausklinikum Hetzelstift Neustadt an der Weinstraße (regionales Traumazentrum).

## Ergebnisse

### Patientenkollektiv und perioperative Parameter

Der Altersmittelwert lag bei 83 ± 8 Jahren (60–102). Es wurden 225/335 Frauen (67 %) und 110/335 Männer (33 %) in die Studie eingeschlossen. 26 von insgesamt 335 operierten Patienten verstarben innerhalb des stationären Aufenthaltes. Dies entspricht einer Klinikmortalität von 7,76 %. In 8 Fällen (30,8 %) wurde die Todesursache lediglich unspezifisch mit der Diagnosestellung „Verschlechterung des Allgemeinzustandes“ klassifiziert. Dies ist sicher zum einen der retrospektiven Datenauswertung geschuldet als auch der Tatsache, dass bei den häufig multimorbiden Patienten auf eine weiterführende Abklärung der Todesursache verzichtet wurde.

Eine detaillierte Darstellung des Patientenkollektivs und der perioperativen Parameter ist in den Tab. 1 und 2 zu finden.

**Table 2:** 

**Operativ**		
Versorgungsart	*Nagelosteosynthese*	*159/335 (48* *%)*
	Nagel, 180 mm	86/159 (54 %)
	Nagel, 200 mm	56/159 (35 %)
	Nagel, > 200 mm	17/159 (12 %)
	Geschlossene Reposition	125/159 (79 %)
	Offene Reposition	34/159 (21 %)
	*Endoprothese*	*158/335 (47* *%)*
	Hemiendoprothese	71/158 (45 %)
	Zementfreie H‑TEP, PM	40/158 (25 %)
	Zementfreie H‑TEP, CC	24/158 (15 %)
	Zementierte H‑TEP	18/158 (12 %)
	Hybride H‑TEP	5/158 (3 %)
	*Schraubenosteosynthese*	*4/335 (1* *%)*
	*Andere Verfahren*	*14/335 (4* *%)*
Dauer der Operation (min)	Mean ± SD (Range)	58 ± 26 min (14–217)
**Postoperativ**		
Postoperative Belastung	Teilbelastung	90 Patienten (27 %)
	Vollbelastung	245 Patienten (73 %)
Dauer des stationären Aufenthalts (Tage)	Mean ± SD (Range)	14 ± 7 Tage (2–72)
Gabe von Erythrozytenkonzentraten	Ja	127/335 (38 %)
	Nein	208/335 (62 %)
Komplikationen	Allgemein	142 (42 %)
	Spezifisch chirurgisch	43 (12 %)
Allgemeine Komplikationen	Harnwegsinfektion	93/142 (65 %)
	Hypokaliämie	31/142 (22 %)
	Pneumonie mit respiratorischer Insuffizienz	24/142 (17 %)
	Nierenversagen	15/142 (11 %)
	Delir	8/142 (6 %)
	Diarrhö (clostridien- oder norovirusassoziiert)	7/142 (5 %)
	Kardiale Dekompensation	4/142 (3 %)
	Hyponatriämie	2/142 (1 %)
Spezifische chirurgische Komplikationen	WHS, Hämatom oder Hämatoserom	11/43 (26 %)
	Periprothetische Fraktur	13/43 (30 %)
	Periprothetische Infektion	10/43 (23 %)
	Luxation	6/43 (14 %)
	Sekundäre Dislokation	4/43 (9 %)
	Implantatversagen	1/43 (2 %)
Folgeeingriffe	–	36/335 (10,75 %)
Intensivtherapie	Ja	51 (15 %)
	Nein	284 (85 %)
Gesamtmortalität	–	26/335 (7,76 %)
Todesursache	Verschlechterung des Allgemeinzustands	8/26
	Nierenversagen	4/26
	Multiorganversagen	3/26
	Urosepsis	3/26
	Lungenarterienembolie, „Critical-illness“-Polyneuropathie, respiratorische Insuffizienz, Nicht-ST-Hebung-Myokardinfarkt, kardiale Dekompensation, Ileus, gastrointestinale Blutung, Leberversagen	Jeweils 1/26

*SD* „standard deviation“, *H‑TEP* Hüfttotalendoprothese, *PM* Metall-Polyethylen-Gleitpaarung, *CC* Keramik-Keramik-Gleitpaarung, *WHS* Wundheilungsstörung

### Zusammenhang der erfassten Patientencharakteristika und der Mortalität

Eine postoperativ auftretende spezifische chirurgische Komplikation erhöhte die Mortalität von 7,5 auf 9,3 % (OR 1,2587; 95%-KI [0,4119–3,8464]; *p* = 0,6864). Hier war die höchste Mortalität bei den Patienten mit einer postoperativen periprothetischen Infektion (20 %) zu nennen. Es fand sich kein Einfluss der allgemeinen Komplikationen auf die Klinikmortalität. Bezogen auf die Vorerkrankungen zeigte sich die höchste Mortalitätsrate bei den multimorbiden Patienten mit 5 Vorerkrankungen (Mortalität von 30,8 %). Patienten mit mehr als 3 Vorerkrankungen hatten ein 10-fach höheres Risiko zu versterben (OR 10,61; 95%-KI [3,681–27,501]; *p* < 0,0001). Bei der Analyse der Mortalität bei den unter Einnahme von gerinnungshemmenden Medikamenten operierten Patienten war eine 6,2-fach erhöhte Mortalitätsrate festzustellen (OR 6,19; 95%-KI [2,69–14,24]; *p* < 0,0001). Bei den Patienten, die eine postoperative intensivmedizinische Behandlung benötigten, lag die Mortalität bei 23,5 % im Vergleich zu den restlichen Patienten, bei welchen die Mortalität deutlich niedriger bei 4,9 % lag. Ein postoperativ intensivpflichtiger Patient hatte eine 5,9-fache Wahrscheinlichkeit zu versterben als ein Patient, der postoperativ keine intensivmedizinische Behandlung benötigte (OR 5,9; 95%-KI [2,56–13,76]; *p* < 0,0001).

Eine detaillierte Darstellung der Korrelationen der erfassten Patientencharakteristika und Mortalität ist im Zusatzmaterial online zu finden.

### Zusammenhang der erfassten Patientencharakteristika und den Komplikationen

Die Patientencharakteristika und deren Einfluss auf das Auftreten von Komplikationen wurden analysiert.

Im Vergleich zu den endoprothetisch versorgten Patienten bzw. Patienten mit einer Schenkelhalsfraktur konnte bei Nagelosteosynthesen (OR 2,2526; 95 %-KI [1,4241–3,5633]; *p* = 0,0005) bzw. pertrochantären Frakturen (OR 2,3766; 95 %-KI [1,5055–3,7519]; *p* = 0,0002) eine Erhöhung der Rate an allgemeinen Komplikationen verzeichnet werden. Die Korrelationen mit den einzelnen spezifischen und allgemeinen Komplikationen waren bis auf eine höhere Rate an Harnwegsinfektionen bei Endoprothesen (*p* < 0,0001) bzw. Schenkelhalsfrakturen (*p* < 0,0001) nicht statistisch signifikant. Die Einnahme von gerinnungshemmenden Medikamenten (OR 1,6585; 95 %-KI [1,0574–2,6013]; *p* = 0,0276), Gabe von Erythrozytenkonzentraten (OR 1,7059; 95 %-KI [1,0970–2,6528]; *p* = 0,0177) und Notwendigkeit einer postoperativen intensivmedizinischen Behandlung (OR 2,0443; 95 %-KI [1,1200–3,7313]; *p* = 0,0198) erhöhten ebenfalls die Rate an allgemeinen Komplikationen. Bei der Analyse der Korrelationen der einzelnen Komplikationen zeigt sich ein statistisch relevanter Einfluss der Notwendigkeit einer postoperativen intensivmedizinischen Behandlung auf das Auftreten von Pneumonien mit respiratorischer Insuffizienz (*p* = 0,0007), Nierenversagen (*p* = 0,0019) und Delir (*p* = 0,0118). Bei den restlichen Parametern konnte kein signifikanter Einfluss festgestellt werden.

Eine detaillierte Darstellung der Korrelationen der erfassten Patientencharakteristika und des Auftretens von Komplikationen ist im Zusatzmaterial online zu finden.

## Diskussion

### Strukturelle und klinikbezogene Merkmale

Hüftgelenknahe Femurfrakturen (HF) stellen eine große Belastung für älteren Patienten dar. Es ist nicht verwunderlich, dass der Beschluss des Gemeinsamen Bundesausschusses (GBA) die Versorgung solcher Frakturen innerhalb von 24 h nach Aufnahme fordert [8]. Diese gesetzliche Vorgabe führte zusammen mit der Umstellung des Krankenhausfinanzierungssystems auf das DRG-Fallpauschalensystem zu einer Steigerung des ökonomischen Drucks und einer Verschärfung der oftmals vorbestehenden strukturellen Defizite in Krankenhäusern [15].

So scheinen neben patientenbezogenen Faktoren auch klinikbezogene Kriterien, die wesentlich an der Infra- und Prozessstruktur der Häuser bedingt sind, Einfluss auf die Mortalität zu haben. Unter den wichtigsten klinikbezogenen Merkmalen, die in der Regel mit einer Erhöhung der Mortalität assoziiert werden, sind mangelnde interne Leitlinien und „standard operating procedure“ (SOP) zur Regelung der Abläufe, Nichtverfügbarkeit von medizinischem Personal und die fehlende operative Kapazität zu erwähnen. Die direkten Folgen solcher Defizite sind Verzögerungen des Operationszeitpunktes und eine Verlängerung der Zeitspanne zwischen Aufnahme und Operation [13, 22]. So konnten Sund et al. [24] zeigen, dass die strukturell klinikbezogenen organisatorischen Defizite eine klare Assoziation mit der Verzögerung der Operation und Mortalität aufwiesen.

Griffiths et al. [13] greifen das allgegenwärtige Problem des „Personalmangels“ auf. Die Autoren konnten belegen, dass eine geringere Personalausstattung an examinierten Krankenpflegern und eine höhere Anzahl von Aufnahmen pro Krankenpfleger mit einem erhöhten Sterberisiko verbunden waren.

Die operative Kapazität wurde in der Arbeit von Ruffing et al. [22] untersucht. Hier wurde in 13,3 % der HF mit einer Verzögerung der operativen Versorgung > 48 h eine logistische Ursache im Sinne von u. a. einer fehlenden OP-Kapazität gefunden. Die Analyse der Daten des strukturierten Dialogs der externen Qualitätssicherung belegten des Weiteren, dass diese Verzögerungen in Rheinland-Pfalz mit 40 % die Kliniken der Regelversorgung und nur mit 15 % die Maximalversorger betrafen [22].

### Patientenbezogene Parameter

Der Altersmittelwert im Kollektiv der operierten Patienten lag ähnlich zu Daten aus der Literatur bei 83 Jahren [20]. In der Querschnittsuntersuchung von Padrón-Monedero et al. [20] wurde der Einfluss von Alter auf die Klinikmortalität bei 31.884 Patienten (Altersmittelwert 83,8 Jahre) mit einer HF analysiert. Hier zeigte sich eine Erhöhung der Klinikmortalität bei Patienten > 85 Jahre (OR 4,14; 95%-KI [3,21–5,34]; *p* < 0,001) sowie bei Patienten 75 bis 84 Jahre (OR 2,08; 95%-KI [1,60–2,70]; *p* < 0,001) gegenüber Patienten < 75 Jahre. Diese Ergebnisse zeigen sich höher als die Ergebnisse der vorliegenden Studie.

Die weibliche Dominanz des Patientenkollektivs sowie das in der vorliegenden Studie beobachtete höhere Mortalitätsrisiko bei dem männlichen Geschlecht können auch in vergleichbaren Studien beobachtet werden [18]. Von Friesendorff et al. [26] untersuchten diesen Zusammenhang in einem Maximalversorger (Bettenzahl 1750) bei 1013 Patienten mit einer HF und beschrieben eine Erhöhung der Mortalität bei männlichem Geschlecht um das 1,5-Fache.

Bergeron et al. [6] untersuchten in einer Studie von 2006 den Zusammenhang mit dem Zeitpunkt der Operation bei 977 Patienten mit HF in einer Klinik der Maximalversorgung (Bettenzahl 571). Hier zeigte sich keine signifikante Erhöhung. In der vorliegenden Studie zeigt sich eine Erhöhung des Mortalitätsrisikos bei der Versorgung > 24 h bzw. 48 h nach Aufnahme, allerdings ohne statistische Signifikanz.

Das postoperative Delir repräsentierte die fünfthäufigste Komplikation im untersuchten Patientenkollektiv mit 6 % (8/142). Wie die Literatur eindeutig belegt, gelten eine präoperativ eingeschränkte kognitive Funktion und fortgeschrittenes Alter als die 2 wichtigsten Risikofaktoren für die Entstehung eines postoperativen Delirs [7]. Unterstützt wird dies durch die Charakteristika des Patientenkollektivs der vorliegenden Studie, in der der Altersmittelwert bei 83 Jahren und der Anteil an Patienten mit einer präoperativ bekannten vordiagnostizierten kognitiven Funktionsstörung (hier konkret einer Demenz) bei 19 % (64/335) lag. Demgegenüber lag die Rate an postoperativ dokumentierten Deliren lediglich bei 6 % und ist höchstwahrscheinlich unterrepräsentiert. Dies kann entweder 1. in einer unvollständigen Dokumentation oder 2. einem unzureichenden ärztlichen/pflegerischen Bewusstsein in der Diagnosestellung begründet sein. Während Punkt 1 aufgrund der Erlösrelevanz aus Autorensicht eher zu vernachlässigen ist, spiegelt Punkt 2 die Notwendigkeit einer orthopädisch/unfallchirurgisch-geriatrischen Zusammenarbeit wider.

Die steigende Mortalität bei höherem CCI und Multimorbidität korreliert mit den Ergebnissen ähnlicher Studien in der Literatur. Padrón-Monedero et al. [20] belegten bei Patienten mit ≥ 3 Vorerkrankungen ein 2,6-fach erhöhtes Mortalitätsrisiko (OR 2,62; 95%-KI [2,38–2,90]); deutlich niedriger als die Ergebnisse dieser Studie.

Diese Ergebnisse sind die Grundlage dafür, dass bereits in vielen Kliniken eine interdisziplinäre Zusammenarbeit zwischen Unfallchirurgen und Geriatern etabliert wurde. Die „AltersTraumaZentren“ sollen durch die interdisziplinäre Herangehensweise die entsprechenden Bedürfnisse, die diese Patienten benötigen, adressieren. Dies umfasst die Mitbehandlung von der Aufnahme über die präoperative Vorbereitung bis hin zur postoperativen Optimierung [21]. Bei Eignung und Erfüllung der logistischen und patientenspezifischen Voraussetzungen kann auch im Rahmen dieser Zusammenarbeit eine geriatrische Frührehabilitation bzw. geriatrische Komplexbehandlung (GKB) eingeleitet werden, um eine Rekonditionierung zu gewährleisten, bis die Rehabilitationsfähigkeit gegeben ist [1]. In der Literatur konnte der positive Effekt solcher Rehabilitationsprogramme nach HF nicht nur auf die Selbstständigkeit, sondern auch auf den gesamten soziofunktionellen Zustand bereits belegt werden [3]. Bestätigend ist eine Beobachtungsstudie von Rapp et al. [21] mit 58.000 Patienten aus 828 Krankenhäusern, die den statistisch signifikanten positiven Effekt eines unfallchirurgisch-geriatrischen Komanagements auf die Mortalität zeigt.

Auch in Ermangelung eines präoperativen Funktionsassessments ist davon auszugehen, dass eine Vielzahl der Patienten auch in unserem Patientenkollektiv bereits vor der Verletzung unter einer eingeschränkten kognitiven Funktion und einem geringen Aktivitätsniveau im Bereich der Aktivitäten des täglichen Lebens (ADL) gelitten hat. Diese Punkte zählen zu den prognostischen Faktoren für ein schlechtes Outcome bei HF [9, 10] und werden in der Regel im Rahmen des geriatrischen Screenings systematisch erfasst. Fukui et al. [11] konnten zeigen, dass das präoperative ADL und Komorbiditäten, inkl. kognitiv-einschränkende Krankheiten, eine entscheidende Rolle für das funktionelle Outcome 6 und 12 Monate nach der operativen Versorgung einer HF spielen.

In der vorliegenden Studie zeigte sich eine Erhöhung des Mortalitäts- und Komplikationsrisikos bei den Patienten, die unter Einnahme von Antikoagulanzien operiert wurden. Die erhöhte Rate an allgemeinen Komplikationen nach HF wurde in der Arbeit von Hoerlyck et al. [14] von 2019 ebenfalls festgestellt. Taranu et al. [25] zeigten eine Korrelation zwischen der Verschiebung der Operation und der Einnahme von Antikoagulanzien und in der Folge einer höheren Mortalität. In diesem Kontext könnte ein einheitlicher Algorithmus zum Umgang mit Antikoagulanzien wesentlich dazu beitragen, die Komplikations- und Mortalitätsrate zu reduzieren.

Eine Korrelation zwischen der Gabe von Erythrozytenkonzentraten und der Mortalität konnte in der vorliegenden Arbeit nicht festgestellt werden. Jedoch wurde ein statistisch relevanter Einfluss auf das Auftreten von allgemeinen Komplikationen beobachtet. Diese Korrelation entspricht den Ergebnissen der Arbeit von Arshi et al. [2] von 2020.

In der vorliegenden Arbeit zeigte sich eine Erhöhung des Mortalitäts- und Komplikationsrisikos bei den Patienten, die eine postoperative intensivmedizinische Behandlung benötigten. Dies korreliert mit den Ergebnissen von Gibson et al. [12]. Muhm et al. [17] stellten ebenfalls fest, dass ein verlängertes präoperatives Zeitintervall bis zur Operation sowie die höhere Anzahl an Nebenerkrankungen häufiger eine Intensivtherapie bedingen. Jedoch wurde eine direkte Korrelation zwischen Mortalität und Intensivtherapie nicht festgestellt.

In der Literatur ist keine Arbeit zu finden, die den Zusammenhang zwischen Einnahme von Antikoagulanzien oder der Notwendigkeit einer postoperativen intensivmedizinischen Behandlung und Mortalität untersucht.

Eine Zusammenfassung der Vergleiche der Klinikmortalität anhand der aktuellen Literatur ist im Zusatzmaterial online zu finden.

### Limitationen

Eine der Einschränkungen dieser Arbeit ist die retrospektive Datenerhebung. Dies verringert die Verlässlichkeit der erhobenen Daten und schränkt insbesondere den Beobachtungszeitraum, der hier auf den stationären Aufenthalt beschränkt bleibt, gegenüber prospektiven Studien ein. Der Vergleich mit den Daten der externen Qualitätssicherung und den Routinedaten der Krankenkassen sollte hier zurate gezogen werden.

Eine andere Limitation der Arbeit sind die teilweise fehlenden Korrelationen zwischen den klinikbezogenen Parametern in der untersuchten Klinik in der vorliegenden Studie und den in Maximalversorgungseinrichtungen, da der primäre Fokus dieser Studie nicht auf dem Vergleich der infrastrukturellen bzw. prozessbedingten Faktoren der unterschiedlichen Versorgungsstufen beruht.

## Fazit für die Praxis

Die vorliegende Studie zeigte einen Zusammenhang zwischen erhöhter Klinikmortalität und

- fortgeschrittenem Alter,
- männlichem Geschlecht,
- Schenkelhalsfrakturen bzw. der Notwendigkeit der Implantation einer Frakturendoprothese,
- dem Auftreten von postoperativen Komplikationen,
- der Frakturversorgung später als 24 h nach Aufnahme.

Ein statistisch signifikanter Zusammenhang bestand zwischen der Klinikmortalität und

- der Anzahl der Komorbiditäten bzw. dem Charlson Comorbidity Index (CCI),
- der Einnahme von Antikoagulanzien,
- der Notwendigkeit einer intensivmedizinischen Behandlung

bei Patienten mit hüftgelenknahen Frakturen an einem regionalen Traumazentrum.

Im Vergleich zu Daten aus Maximalversorgungskliniken zeigt sich diese Mortalität bei multimorbiden Patienten (≥ 3 Komorbiditäten) 5‑fach höher.

## Supplementary Information





## References

[1] Borchelt M, Pientka L, Wrobel N (2004). Abgrenzungskriterien der Geriatrie.

[2] Arshi A, Lai WC, Iglesias BC (2020). Blood transfusion rates and predictors following geriatric hip fracture surgery. Hip Int.

[3] Bachmann S, Finger C, Huss A (2010). Inpatient rehabilitation specifically designed for geriatric patients: systematic review and meta-analysis of randomised controlled trials. BMJ.

[4] Ministerium für Soziales und Intgration Baden-Württemberg (2019). Krankenhausplan 2019 Baden-Württemberg.

[5] Bäuerle D, Specht-Leible N, Voß E (2004). Veränderungen des Hilfe- und Pflegebedarfs nach hüftnahen Frakturen im höheren Lebensalter. Z Gerontol Geriatr.

[6] Bergeron E, Lavoie A, Moore L (2006). Is the delay to surgery for isolated hip fracture predictive of outcome in efficient systems?. J Trauma.

[7] Bickel H, Gradinger R, Kochs E (2004). Incidence and risk factors of delirium after hip surgery. Psychiatr Prax.

[8] Gemeinsamer Bundesausschuss (2019). Beschluss des Gemeinsamen Bundesausschusses über eine Richtlinie zur Versorgung der hüftgelenknahen Femurfraktur.

[9] Cornwall R, Gilbert MS, Koval KJ (2004). Functional outcomes and mortality vary among different types of hip fractures: a function of patient characteristics. Clin Orthop Relat Res.

[10] Eastwood EA, Magaziner J, Wang J (2002). Patients with hip fracture: subgroups and their outcomes. J Am Geriatr Soc.

[11] Fukui N, Watanabe Y, Nakano T (2012). Predictors for ambulatory ability and the change in ADL after hip fracture in patients with different levels of mobility before injury: a 1-year prospective cohort study. J Orthop Trauma.

[12] Gibson AA, Hay AW, Ray DC (2014). Patients with hip fracture admitted to critical care: epidemiology, interventions and outcome. Injury.

[13] Griffiths P, Maruotti A, Recio Saucedo A (2019). Nurse staffing, nursing assistants and hospital mortality: retrospective longitudinal cohort study. BMJ Qual Saf.

[14] Hoerlyck C, Ong T, Gregersen M (2020). Do anticoagulants affect outcomes of hip fracture surgery? A cross-sectional analysis. Arch Orthop Trauma Surg.

[15] Lüngen M, Lauterbach KW (2003). DRG in deutschen Krankenhäusern: UmSetzung und Auswirkungen.

[16] Muhm M, Arend G, Ruffing T (2013). Mortality and quality of life after proximal femur fracture—effect of time until surgery and reasons for delay. Eur J Trauma Emerg Surg.

[17] Muhm M, Walendowski M, Danko T (2014). Einflussfaktoren auf den stationären Verlauf von Patienten mit hüftgelenknahen Femurfrakturen. Z Gerontol Geriat.

[18] Müller-Mai CM, Schulze Raestrup US, Kostuj T (2015). Einjahresverläufe nach proximalen Femurfrakturen. Unfallchirurg.

[19] Novack V, Jotkowitz A, Etzion O (2007). Does delay in surgery after hip fracture lead to worse outcomes? A multicenter survey. Int J Qual Health Care.

[20] Padrón-Monedero A, López-Cuadrado T, Galán I (2017). Effect of comorbidities on the association between age and hospital mortality after fall-related hip fracture in elderly patients. Osteoporos Int.

[21] Rapp K, Becker C, Todd C (2020). The association between orthogeriatric co-management and mortality following hip fracture. Dtsch Arztebl Int.

[22] Ruffing T, Haunschild M, Egenolf M (2016). Verzögerte Versorgung hüftgelenknaher Femurfrakturen. Unfallchirurg.

[23] Smektala R, Grams A, Pientka L (2008). Leitlinie oder Landrecht bei der Versorgung der Schenkelhalsfraktur? Eine Analyse der Versorgungssituation in Nordrhein-Westfalen. Dtsch Arztebl Int.

[24] Sund R, Liski A (2005). Quality effects of operative delay on mortality in hip fracture treatment. Qual Saf Health Care.

[25] Taranu R, Redclift C, Williams P (2018). Use of anticoagulants remains a significant threat to timely hip fracture surgery. Geriatr Orthop Surg Rehabil.

[26] Von Friesendorff M, McGuigan FE, Wizert A (2016). Hip fracture, mortality risk, and cause of death over two decades. Osteoporos Int.

