# Supplementary material for: Risk factors affecting mortality in patients with hip fractures at a regional trauma center
Source: Z Gerontol Geriatr. 2021 Mar 23;54(6):561–70. [Article in German] doi: 10.1007/s00391-021-01869-9 (PMC8458164; doi:10.1007/s00391-021-01869-9)
Supplement: Supplementary file 1 [file 391_2021_1869_MOESM1_ESM.docx]

Tabelle 3: Statistische Korrelation der erfassten Patientencharakteristika und Mortalität

| **Risikofaktor** | **Klinikmortalität**  **(Odds Ratio)** | **Signifikanz**  **(p-Wert)** | **Konfidenz-Intervall** |
| --- | --- | --- | --- |
| Alter 75-84 vs. < 75 | 2,617 | 0,3747 | [0,313 - 21,901] |
| Alter ≥ 85 vs. < 75 | 5,126 | 0,1167 | [0,665 - 39,498] |
| Männliches Geschlecht | 1,85 | 0,0555 | [0,82 - 4,14] |
| Versorgung mit Endoprothese vs. Nagelosteosynthese | 1,2 | 0,1942 | [0,523 - 2,780] |
| Schenkelhalsfraktur vs. pertrochantäre Fraktur | 1,35 | 0,0555 | [0,6 - 3,02] |
| Operationszeitpunkt  24-48h vs. <24h | 1,896 | 0,2341 | [0,661 - 5,441] |
| Operationszeitpunkt  >48h vs. <24h | 2,217 | 0,1792 | [0,694 - 7,086] |
| Operationszeitpunkt  24-48h vs. >48h | 1,1692 | 0,8275 | [0,2865 - 4,7712] |
| Gabe Erythrozytenkonzentrate | 1,04 | 0,9258 | [0,46 - 2,37] |
| Komorbiditäten ≥3 vs. <3 | 10,61 | **< 0,0001*** | **[3,681 - 27,501]** |
| Einnahme gerinnungshemmender Medikation | 6,19 | **< 0,0001*** | **[2,69 - 14,24]** |
| Notwendigkeit einer postoperativen intensivmedizinischen Behandlung (ja vs. nein) | 5,9 | **< 0,0001*** | **[2,56 - 13,76]** |

*statistisch signifikant

| Tabelle 4: Statistische Korrelation der erfassten Patientencharakteristika und Komplikationen | | | | | | |
| --- | --- | --- | --- | --- | --- | --- |
| **Risikofaktor** | **Allgemeine Komplikation (Odds Ratio)** | **Signifikanz**  **(p-Wert)** | **Konfidenz-Intervall** | **Spezifische Komplikation** | **Signifikanz**  **(p-Wert)** | **Konfidenz-Intervall** |
| Alter 75-84 vs. < 75 | 1,0009 | 0,9978 | [0,5180 - 1,9340] | 1,0582 | 0,9048 | [0,4184 - 2,6767] |
| Alter ≥ 85 vs. < 75 | 1,3202 | 0,3841 | [0,7062 - 2,4681] | 1,5594 | 0,3527 | [0,6110 - 3,9805] |
| Männliches Geschlecht | 0,7104 | 0,1391 | [0,4515 - 1,1176] | 1,3015 | 0,4299 | [0,6765 - 2,5040] |
| Versorgung mit Nagelosteosynthese vs. Endoprothese | 2,2526 | **0,0005*** | **[1,4241 - 3,5633]** | 0,8328 | 0,5937 | [0,4253 - 1,6309] |
| Pertrochantäre Fraktur vs. Schenkelhalsfraktur | 2,3766 | **0,0002*** | **[1,5055 - 3,7519]** | 0,8240 | 0,5683 | [0,4237 - 1,6024] |
| Operationszeitpunkt  24-48h vs. <24h | 0,7988 | 0,5196 | [0,4032 - 1,5826] | 0,9049 | 0,8454 | [0,3313 - 2,4712] |
| Operationszeitpunkt  >48h vs. <24h | 0,6700 | 0,2386 | [0,3442 - 1,3042] | 0,7945 | 0,6515 | [0,2928 - 2,1558] |
| Operationszeitpunkt  24-48h vs. >48h | 0,8387 | 0,6970 | [0,3460 - 2,0329] | 0,8780 | 0,8466 | [0,2350 - 3,2802] |
| Gabe Erythrozytenkonzentrate | 1,7059 | **0,0177*** | **[1,0970 - 2,6528]** | 1,2477 | 0,5070 | [0,6489 - 2,3991] |
| Komorbiditäten ≥3 vs. <3 | 1,4102 | 0,1434 | [0,8898 - 2,2349] | 0,9557 | 0,8964 | [0,4829 - 1,8912] |
| Einnahme gerinnungshemmender Medikation | 1,6585 | **0,0276*** | **[1,0574 - 2,6013]** | 1,0977 | 0,7804 | [0,5698 - 2,1148] |
| Notwendigkeit einer postoperativen intensivmedizinischen Behandlung (ja vs. nein) | 2,0443 | **0,0198*** | **[1,1200 - 3,7313]** | 1,5756 | 0,2678 | [0,7050 - 3,5212] |

*statistisch signifikant

Tabelle 5: Zusammenfassung Vergleich der Klinikmortalität anhand der aktuellen Literatur

| **Parameter** | **Klinikmortalität (Odds Ratio)** | |
| --- | --- | --- |
|  | **Regionales Traumazentrum** | **Vergleichbare Studien*** |
| Alter 75-84 vs. < 75 | 2,617 (p = 0,3747) | 2,08 (p < 0,001) [2] |
| Alter ≥ 85 vs. < 75 | 5,126 (p = 0,1167) | 4,14 (p < 0,001) [2] |
| Männliches Geschlecht | 1,75 (p = 0,1352)** | 1,5 (p < 0,001) [3]** |
| Operationszeitpunkt  24-48h vs. <24h  > 48h vs. <24h | 1,896 (p = 0,2341)  2,217 (p = 0,1792) | 0,88 (p = 0,2) [1]  1,16 (p = 0,2) [1] |
| Komorbiditäten ≥ 3 | **10,61 (p < 0,0001)** | 2,62 (p < 0,001) [2] |

* Literatur-Daten aus Kliniken der Maximalversorgung bzw. Universitätskliniken oder überregionalen Traumazentren sowie aus Multizentren und Querschnittsstudien

** Relative risk
